# Pediatric Elbow Dislocations and Associated Fractures

**DOI:** 10.3390/children10060993

**Published:** 2023-06-01

**Authors:** Laura Lewallen, Marilyn E. Elliott, Amy McIntosh, Christine A. Ho

**Affiliations:** 1 Department of Orthopaedic Surgery, University of Chicago Medicine, Chicago, IL 60637, USA; 2 Scottish Rite for Children, Dallas, TX 75219, USA; 3 Department of Orthopaedic Surgery, Children’s Medical Center of Dallas, Dallas, TX 75235, USA; 4 Department of Orthopaedic Surgery, University of Texas Southwestern, Dallas, TX 75390, USA

**Keywords:** elbow dislocation, medial epicondyle fracture, pediatric elbow injury

## Abstract

The objective was to evaluate pediatric patients with acute elbow dislocation and/or associated fracture to determine which were indicated for surgical intervention, using a single institution, Institutional Review Board (IRB) approved retrospective review of patients who presented to the Emergency Department (ED) with an acute elbow dislocation. Inclusion criteria were age ≤ 18 years, acute elbow dislocation injury, and appropriate imaging. A total of 117 patients were included 37 had a simple elbow dislocation, 80 had an associated fracture (medial epicondyle 59, lateral condyle 9, radial head/neck 7, other 5). A total of 62% (73/117) were male. The average age was 10.3 years (range 4–17). Mechanisms of injury included: falls from height/playground equipment (46), trampoline (14), and sports (57). All 37 patients with a simple elbow dislocation were successfully treated with closed reduction. Of the 80 patients with an associated fracture, 30 (38%) went on to open reduction internal fixation (ORIF). A total of 59 patients had an associated medial epicondyle fracture; 24 (41%) of whom went on to ORIF. Nine patients had an associated lateral condyle fracture, five (56%) of whom went on to ORIF. Patients with a simple elbow dislocation can be successfully treated with a closed reduction in the ED. However, 30/80 patients with an associated fracture (medial epicondyle, lateral condyle, or radial neck) required operative management.

## 1. Introduction

Upper extremity injuries are common in the pediatric population. Elbow dislocations account for approximately 3–6% of elbow injuries in the pediatric population [1,2]. These injuries most often occur in children between 10–15 years of age [2]. Boys have a higher incidence compared to girls, by a ratio of 3:1 [3].

The mechanism of injury typically involves a fall on an outstretched arm, most often with the elbow extended. These types of injuries often occur as a result of a fall from playground equipment, bicycles, trampolines, or sporting activities. Posterolateral displacement is the most frequent pattern of dislocation. A valgus force may result in the avulsion of the ulnar collateral ligament (UCL) and/or an associated medial epicondyle fracture. Direct force may also cause a medial epicondyle fracture [4].

Various types of fractures about the elbow may occur in association with a dislocation event. The majority of associated injuries in the pediatric population involve simple fracture patterns, though more complex fracture patterns do occur [5]. Medial epicondyle fractures are the most common; associated with an elbow dislocation in 30–60% of pediatric cases [2,6]. These fractures account for approximately 12–20% of elbow injuries in the pediatric population [4,7]. Other fractures associated with pediatric elbow dislocations include lateral condyle, radial head/neck, coronoid process, or olecranon.

The standard of treatment for an acute elbow dislocation is urgent closed reduction, followed by brief immobilization and early active range of motion [1,6]. Reduction maneuvers including hyperextension, longitudinal traction, forearm supination, and elbow flexion are usually successful. Patients with an open injury, associated neurovascular injury, or persistent instability typically require open reduction and exploration. Open reduction internal fixation (ORIF) is indicated for those with an incarcerated fracture fragment (i.e., medial epicondyle) [8]. However, controversy exists regarding the amount of fracture displacement which is acceptable for non-operative management [9,10].

Our hypothesis was that a subset of patients with these injuries (elbow dislocation and associated fracture) may be safely managed with reduction/surgery in a delayed fashion, rather than acute reduction in the Emergency Department. The objective of this study was to evaluate pediatric patients with an acute elbow dislocation, and/or associated elbow fracture, to further characterize which patients were indicated for surgical intervention.

## 2. Materials and Methods

### 2.1. Study Design

This was an Institutional Review Board (IRB) approved retrospective review of patients who presented to our Emergency Department (ED) with an acute elbow dislocation between 1 January 2008 and 31 December 2016. The study’s IRB number is STU-092016-039 and was approved on 12 October 2016. The study was performed at a single institution, a pediatric level-1 trauma center.

### 2.2. Population

Patients with an acute elbow injury who were treated with closed reduction were first identified from paper fluoroscopy logs kept by the radiology department. In total, 303 elbow injuries were identified in this manner. The type of elbow injury was confirmed through a chart review of the orthopedic consult notes and radiology reports, as well as a radiographic review of the images.

### 2.3. Inclusion/Exclusion Criteria

Patients were included in the final analysis if they met the following criteria: (1) acute elbow dislocation with or without an associated elbow fracture, (2) age 18 years or younger at the time of injury, and (3) appropriate imaging to characterize the injury. Patients were excluded from the final analysis if they (1) had any acute elbow injury that did not include an elbow dislocation (i.e., radial head dislocation, supracondylar or other distal humerus fracture, Monteggia injury), (2) were over the age of 18 years at the time of injury, or (3) did not have appropriate imaging.

### 2.4. Data Collection/Analysis

The following data were collected: age, gender, laterality, mechanism of injury, treatment at an outside hospital (if any), nature of associated fracture (if any), amount of displacement at the fracture site, whether the closed reduction was attempted, whether surgery was necessary, time from initial treatment (closed reduction) to surgery, indication for surgery, a surgical procedure performed, and length of follow up. Outside treatment was collected from either Emergency Medical Services (EMS) reports or initial ED documentation. The mechanism of injury was grouped into the following categories: fall from height or playground activity, sporting activity, or trampoline. Initial closed reduction data were obtained from the orthopedic consult notes and procedure notes. Surgical indications and procedures performed were determined from the operative notes. The maximum amount of displacement at the fracture site was measured on post-reduction radiographs, for those patients with an associated fracture (medial epicondyle, lateral condyle, radial head/neck. Length of follow-up was collected from the clinic progress notes.

## 3. Results

### 3.1. Demographic Data

**Figure 1:**
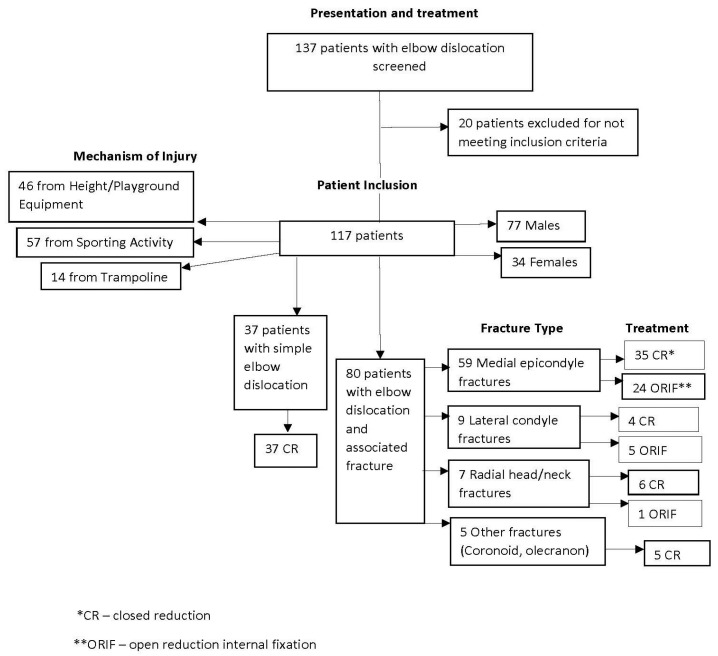
Flow chart of patient presentation and treatment.

In total, 303 patients with acute elbow injuries were identified. A total of 117 met our inclusion criteria. A total of 31.6% (37/117) of patients had a simple elbow dislocation. A total of 68.4% (80/117) had an associated fracture, with the medial epicondyle being the most common (73.8%, 59/80). Nine patients had an associated lateral condyle fracture, seven patients had a radial head/neck fracture, and five patients had other fractures (coronoid, olecranon) (Figure 1). There were more males than females (62.4% versus 37.6%). The average age at the time of injury was 10.3 years (range 4–17 years).

### 3.2. Mechanism of Injury

As shown in Table 1, the most common mechanisms of injury were a fall from height/playground equipment (39.3%, 46/117) or sporting activity (48.7%, 57/117). Trampoline injuries were less common (12%, 14/117).

### 3.3. Treatment

All 117 patients underwent closed reduction under sedation in the ED. Reductions were performed by resident physicians or advanced practice providers (APPs), including orthopedic nurse practitioners and orthopedic physician assistants. Standard closed reduction maneuvers were performed, including a combination of longitudinal traction, supination, and elbow flexion, anteriorly directed force on the olecranon.

The 37 patients with a simple elbow dislocation (i.e., no associated fractures) were all successfully treated with closed reduction.

**Figure 2:**
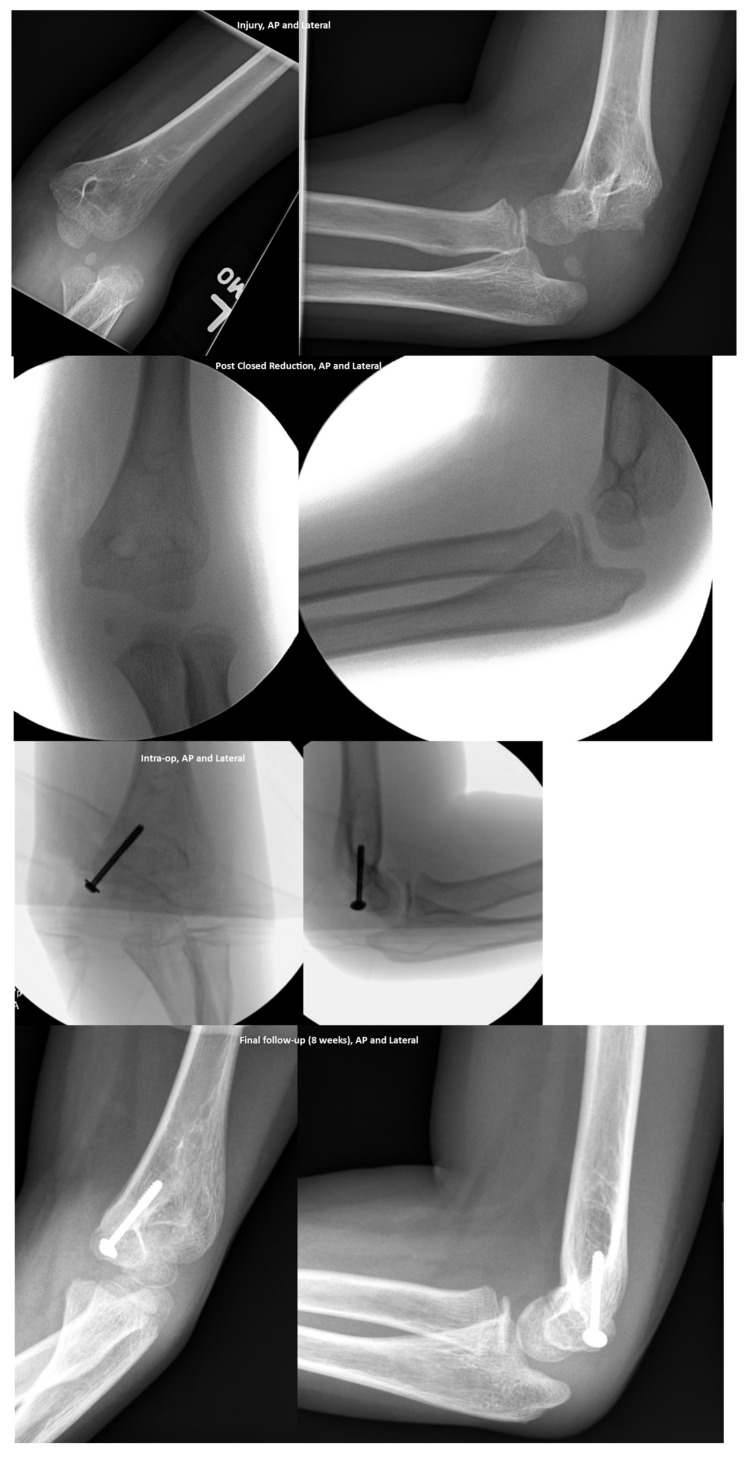
The clinical course of a 9-year-old with a medial epicondyle fracture. Injury (first row from the top, left image—Antero-Posterior (AP); right image—Lateral); Post Closed Reduction (second row from the top, left image—AP scan; right image—Lateral); Intra-operative (third row from the top, left image—AP; right image—Lateral); Final follow up at 8 weeks (fourth from the top/Bottom row, left image—AP; right image—Lateral).

The number of associated fractures, as well as the treatment summary, are shown in Table 2. Of the 80 patients with an associated fracture, 73.8% (59/80) had a medial epicondyle fracture. A total of 40.7% (24/59) of the medial epicondyle fractures were treated with open reduction internal fixation after reduction of the elbow dislocation (Figure 2).

The indication for surgery was the amount of fracture displacement per the attending surgeon’s operative notes in 79.2% (19/24), and an incarcerated fracture fragment in 20.8% (5/24).

**Figure 3:**
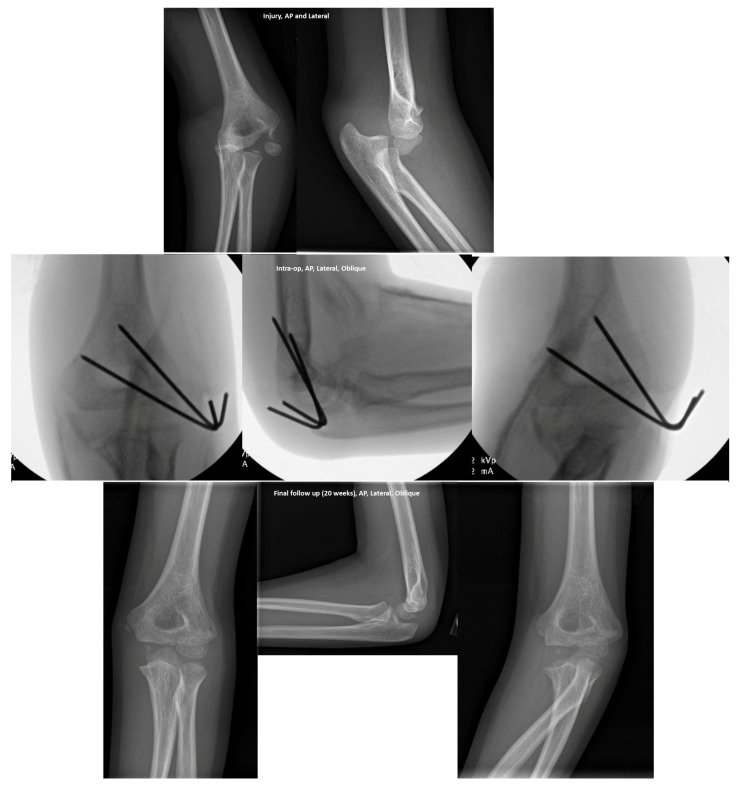
The clinical course of a 7-year-old male with a lateral condyle fracture. Injury (1st row from the top, left image—Antero-Posterior (AP); right image—Lateral); Intra-Operative (2nd row from the top, left image—AP; middle image—Lateral; right image—Oblique); Final follow-up at 20 weeks (3rd row from top/bottom row, left image—AP; middle image—Lateral; right image—Oblique.

Nine patients had a lateral condyle fracture. A total of 55.6% (5/9) of these patients underwent open reduction internal fixation after reduction of the elbow dislocation (Figure 3).

The indication for surgery in all five patients was the amount of fracture displacement. One patient was treated with closed reduction in the operating room (OR), due to ongoing instability after initial reduction in the ED.

Seven patients had a radial head/neck fracture. One of these patients was treated with open reduction internal fixation, due to displacement of the fracture fragment.

Five patients had various other associated fractures (coronoid, olecranon). None of these patients were treated surgically.

For those patients with an associated fracture (medial epicondyle, lateral condyle, or radial head/neck), the maximum amount of displacement was measured on post-reduction radiographs. Several patients did not have appropriate imaging to measure the maximum displacement. Comparisons were made between patients treated non-operatively and those treated with ORIF. The results are shown in Table 3.

Of all patients with an associated fracture in which maximum displacement measurements could be made (*n* = 51), there was a significant difference (*p* = 0.0004) between those treated non-operatively (mean displacement = 4.3 mm) and those treated with ORIF (mean displacement = 10.58 mm). For patients with a medial epicondyle fracture, there was also a statistically significant difference (*p* = <0.0001) between those treated non-operatively (mean displacement = 4.30 mm) and those treated with ORIF (mean displacement = 12.85 mm). The lateral condyle and radial head/neck groups were too small to make comparisons or draw conclusions regarding fracture displacement.

In summary, 37.5% (30/80) of patients with an associated fracture went on to ORIF. Over 40% of patients with an associated medial epicondyle fracture, and over 50% of those with an associated lateral condyle fracture underwent operative management.

All surgeries were performed by fellowship-trained pediatric orthopedic surgeons. The average time from initial treatment (closed reduction) to surgery was 2.8 days (range 0–13 days, median 1 day). Of the 30 patients who were treated with ORIF, 28 had surgery within 7 days of closed reduction. The other two patients were initially managed non-operatively, then found to have a further displacement at the initial follow up visit, prompting the decision for surgical intervention (at 11 days and 13 days, respectively).

The average follow-up in this series was 65 days (range 0–666 days, median 46 days).

## 4. Discussion

In our series, none of the patients who presented with a simple elbow dislocation required operative intervention. These findings suggest that these injuries may be treated successfully with closed reduction under sedation in the ED.

Elbow dislocations in the pediatric population are a relatively uncommon injury. A recent study by Hyvonen et al. showed the stable incidence of elbow dislocations in the pediatric population in Finland from 1996–2014 [11]. There is consensus regarding the absolute indications for operative intervention, including neurovascular compromise, open injury, incarcerated fracture fragment, or inability to achieve a concentric, stable reduction. However, controversy exists regarding the management of concomitant injuries and the amount of fracture displacement which is amenable to non-operative treatment [7]. The purpose of this study was to evaluate pediatric patients with an acute elbow dislocation, to further characterize which patients were indicated for surgical intervention.

The average age (10.3 years) and gender distribution (63% male) in our cohort were similar to previous reports [2]. Sporting activities and fall from height/playground equipment were the most common mechanisms of injury.

Nearly 70% (80/117) of patients in our study had an associated fracture. Consistent with previous reports [7,8], medial epicondyle fracture was the most commonly associated injury (74%). As previously reported by Silva et al., associated lateral condyle fractures were uncommon [12]. ORIF was performed in nearly 40% of patients with an associated fracture, most commonly medial epicondyle, and lateral condyle fractures.

Murphy et al. recently reported a series of 145 patients with an elbow dislocation and noted concomitant fractures in 80% (114/145) [2]. Medial epicondyle fracture was the most common (60%). In their series, 59% of patients were treated with ORIF of associated fractures. The complication rate was found to be 14% (21/145). The authors identified risk factors for less than excellent functional outcomes including the presence of multiple associated fractures, operative intervention, and prolonged immobilization.

In our series, over 40% of patients with an associated medial epicondyle fracture and over 50% of patients with an associated lateral condyle fracture were treated surgically. Based on our findings, approximately half of the patients will be going on to surgery within the following day (median time to surgery was 1 day). With the assumption that the patient is neurovascularly intact, with a palpable pulse, and comfortably splinted, it may be reasonable to postpone closed reduction under sedation in the ED and proceed with operative intervention in a timely manner rather than subjecting the child to two anesthetics within 24 h. While both moderate sedation as well as general anesthesia in pediatric patients continues to improve in safety, there is evidence in laboratory animals that early exposure to anesthesia is associated with long-term brain changes [13,14], and that in children under the age of 4 years old, multiple, or prolonged exposures to anesthesia may have adverse effects on behavior, learning, and memory [15,16,17]. In addition, adverse events are a known risk of sedation and anesthesia, which may range from mild, such as a failed sedation [18,19], to a more serious adverse effect such as cardiac arrest [20]. While there is still no direct evidence linking anesthesia to negative central nervous system effects in children, exposure to anesthetic agents should be minimized, when possible, in the pediatric age group.

On the other hand, acute reduction in the ED is the standard of care for all joint dislocations. Urgent reduction results in improved patient comfort, decreased swelling and tension on the soft tissues, and therefore decreased risk of developing neurovascular complications. It is reasonable to conclude from our findings, that reduction IS indicated in the acute setting given the high likelihood of success and the possibility that surgery may not be necessary. A decision for surgery may then be made electively. This also makes the timing of surgery less urgent, in the event of OR delays and other scheduling hurdles.

Limitations of this study include its retrospective nature and lack of long-term follow-up, as is common in many studies looking at orthopedic trauma injuries. The study was performed in a single institution and is therefore limited in its generalizability. The main weakness of the study is that the indication for operative intervention for the medial epicondyle fracture varied by surgeon, as the indications for fixation of closed, non-incarcerated medial epicondyle fractures are controversial. Some surgeons in our practice feel strongly that any medial epicondyle fracture with an associated dislocation should be surgically fixed to prevent valgus instability, while others feel the non-operative treatment is appropriate with a displacement of the fragment up to 1 cm, consistent with prior published studies [9].

Future studies assessing other methods of evaluating fracture morphology may be worthwhile for these types of injuries. Computed tomography (CT) scans are used with caution in the pediatric population, but may have a role in some cases.

## 5. Conclusions

In conclusion, patients with a simple elbow dislocation (no associated fracture) can be successfully treated with a closed reduction in the ED. However, nearly half of patients with an elbow dislocation and associated medial epicondyle fracture or lateral condyle fracture go on to secondary operative management. This is important to consider when counseling patients and families regarding the initial management of these injuries, likelihood of surgery, and the possibility of the child having multiple anesthetics. Based on these findings, we recommend urgent reduction in the ED for patients with an associated fracture, with plans for surgery in a timely fashion as indicated.

## Figures and Tables

**Table 1:** Demographic Data.

		*n* = 117
Sex	Male	73
	Female	44
Mechanism of Injury	Fall from height/playground equipment	46
	Sporting Activity	57
	Trampoline	14

**Table 2:** Injury Pattern and Treatment Summary.

	*n*	CR * Attempted?	CR * Successful?	ORIF **	Indication for ORIF **
TOTAL INCLUDED	117	117	85	30	
Simple dislocation	37	37	37	0	
Associated fracture	80	80	50	30	
Medial epicondyle fracture	59	59	35	24 (40.7%)	fracture displacement—19 (79.2%)
					incarcerated fracture fragment—5 (20.8%)
Lateral condyle fracture	9	9	4	5 (55.6%)	fracture displacement—5 (100%)
Radial head/neck fracture	7	7	6	1 (14.3%)	fracture displacement—1
Other (coronoid, olecranon)	5	5	5	0	

CR *—Closed Reduction. ORIF **—Open Reduction Internal Fixation.

**Table 3:** Comparisons of Post-Reduction Maximum Displacement between Open Reduction/Internal Fixation (ORIF) and No-ORIF groups.

Variables	Overall		No-ORIF Group		ORIF Group		*p*-Value
	*n*	Mean ± Std *, Med (Range)	*n*	Mean ± Std *, Med (Range)	*n*	Mean ± Std *, Med (Range)	
Fracture displacement (mm)	51	6.52 ± 5.28, 5.00 (0, 21.10)	33	4.30 ± 3.50, 4.00 (0, 11.00)	18	10.58 ± 5.66, 11.05 (1.50, 21.10)	0.0004
Fracture displacement (mm)—with medial epicondyle fracture	47	6.85 ± 5.37, 6.80 (0, 21.10)	33	4.30 ± 3.50, 4.00 (0, 11.00)	14	12.85 ± 4.07, 12.75 (6.80, 21.10)	<0.0001
Fracture displacement (mm)—with lateral condyle fracture	4	2.63 ± 1.11, 2.50 (1.50, 4.00)	0		4	2.63 ± 1.11, 2.50 (1.50, 4.00)	

Std *—standard deviation.

## Data Availability

The data presented in this study are available on request from the corresponding author. The data are not available publicly due to privacy reasons.
